# Association of the systemic immune-inflammation index with anemia: a population-based study

**DOI:** 10.3389/fimmu.2024.1391573

**Published:** 2024-05-10

**Authors:** Shuying Chen, Jigang Xiao, Wenyu Cai, Xulin Lu, Chenxi Liu, Yan Dong, Yingchun Zheng, Ge Song, Qi Sun, Huijun Wang, Zhijian Xiao

**Affiliations:** ^1^ State Key Laboratory of Experimental Hematology, National Clinical Research Center for Blood Diseases, Haihe Laboratory of Cell Ecosystem, Institute of Hematology and Blood Diseases Hospital, Chinese Academy of Medical Sciences & Peking Union Medical College, Tianjin, China; ^2^ StateTianjin Institutes of Health Science, Tianjin, China

**Keywords:** systemic immune-inflammation index, anemia, NHANES, cross-sectional study, inflammatory factors

## Abstract

**Background:**

Inflammation has been reported to be related to anemia. As a novel inflammatory marker, Systemic immune-inflammation index (SII) has not been studied with Anemia. The aim of this study was to investigate the possible relationship between SII and anemia.

**Methods:**

This retrospective cross-sectional survey was conducted using data from the 2005–2018 National Health and Nutrition Examination Survey (NHANES) population. In total, 19851 American adults aged ≥18 years were included. SII was calculated as the platelet count×neutrophil count/lymphocyte count. Anemia was defined as hemoglobin (Hgb) levels of < 13 g/dL in males and < 12 g/dL in females. Logistic regression analyses, subgroup analyses and sensitivity analyses were performed to investigate the relationship between SII and anemia.

**Results:**

Our study included a total of 19851 patients, of which 1501 (7.6%) had anemia. After adjusting for all covariates, the multivariate logistic regression analysis showed that a higher SII (In-transform) level was associated with increased likelihood of anemia (OR=1.51, 95% CI: 1.36–1.68, P<0.001). The association between SII and anemia exhibited a nonlinear manner. The positive correlation between SII and anemia was related to the severity of anemia. Subgroup analysis showed that there was no significant dependence on age, family income, body mass index, hypertension, kidney disease and cancer except gender on this positive association. Furthermore, sensitivity analyses confirmed the robustness of our results.

**Conclusion:**

Our study demonstrated that SII was positively associated with anemia especially among female participants. And this positive correlation was related to the severity of anemia. Further large-scale prospective studies are still needed to analyze the role of SII in anemia.

## 1 Introduction

Anemia is the most common blood disorder and remains the major global public health problem, which affects more than 30% of the world’s population (1). It is a condition characterized by a lower-than-normal level of red blood cells or hemoglobin in the blood, leading to reduced oxygen-carrying capacity. This can lead to symptoms such as fatigue, weakness, dizziness, and shortness of breath, which can significantly impact an individual’s daily activities, work performance, and overall well-being. It is more common among females and the older population (2). In the United States, anemia is a common condition with multifactorial and complex causes, including nutritional deficiencies, chronic diseases, immune factors and certain medications. Anemia has been associated with an increased risk of a range of health conditions, including cardiovascular diseases, cognitive impairment, and complications during pregnancy. Numerous studies have demonstrated that anemia is associated with an increased risk of mortality, particularly in older adults and individuals with underlying chronic conditions (3). Early identification of anemia provides an opportunity to delay or prevent disease onset and improve treatment outcomes. Jeong-Yeol Park et al. found that the diagnosis and early intervention of anemia in gynecologic cancer patients can decrease the blood transfusion rate within 3 weeks after the operation (4). Therefore, to find a cheap and easy-to-obtain index which is closely related to anemia is very valuable.

Systemic immune-inflammation index (SII) as a stable and accurate indicator of the local or overall immune response and systemic inflammation in the human body, has been confirmed to be related to the prognostic value for tumors and chronic diseases induced by chronic inflammation or immune dysfunction (5–8). This indicator integrated three inflammatory factors, including platelets, neutrophils and lymphocytes (9). In recent years, the application field of SII has been expanding continuously, and more studies have shown that SII can also be used to predict the risk of certain diseases and monitor the effect of treatment.

It is widely recognized that inflammation plays a significant role in the development and persistence of anemia (10). Inflammatory processes can disrupt the body’s normal mechanisms for producing and regulating red blood cells and can contribute to the development of various types of anemia (11). Inflammation can affect the body’s ability to effectively use and regulate iron, which is essential for the production of hemoglobin and red blood cells. Additionally, inflammatory cytokines can suppress the production of erythropoietin, a hormone that stimulates red blood cell production in the bone marrow. At present, the research on the inflammatory factors related to anemia mainly focuses on neutrophil-to-lymphocyte ratio (NLR), cytokines and innate immune cells such as phagocytes and dendritic cells (12, 13). However, it is not yet clear how the inflammatory level indicator SII and anemia are related.

Therefore, to explore the association between SII and anemia among participants in the US National Health and Nutrition Examination Survey (NHANES), we carried out a population-based investigation and assumed that an elevated SII would be associated with a higher risk of anemia.

## 2 Materials and methods

### 2.1 Data sources and study population

NHANES is an American cross-sectional survey in which participants are selected using a multistage, stratified, probability approach, designed to collect nationally representative data from the non-institutionalized Americans (https://www.cdc.gov/nchs/nhanes/). The NHANES collects demographic and in-depth health information via home visits, screening, and laboratory testing conducted by a mobile examination center (MEC). The NHANES was authorized by the National Center for Health Statistics (NCHS) Ethics Review Committee, and all participants complete written informed consent forms before participating. The secondary analysis did not require additional Institutional Review Board approval.

Seven two-year cycles (2005–2018) of data from NHANES were used for our analysis. Participants in our study were over 18 years and had completed interviews and assessments at a MEC. We excluded individuals with missing data on hemoglobin (Hgb) values, SII values, covariates and pregnant women. Finally, this research included a total of 19851 individuals.

### 2.2 Anemia ascertainment

Anemia was defined according to age and sex normal Hgb values using The World Health Organization guidelines (14). For 15 years of age and above males, no anemia is 13g/dL or higher, mild is (11–12.9) g/dL, moderate is (8–10.9) g/dL and severe is lower than 8g/dL. For 15 years of age and above females (non-pregnant), no anemia is 12g/dL or higher, mild is (11–11.9) g/dL, moderate is (8–10.9) g/dL and severe is lower than 8g/dL. An automated hematology analyzing device (the UniCel DxH 800 analyzer) in the NHANES mobile examination center produces a complete blood count from blood specimens, which provided blood cell distributions for all participants. Anemia was treated as an outcome variable in our study.

### 2.3 Definition of SII

Lymphocyte, neutrophil, and platelet counts (expressed as ×10^3^ cells/μl) were measured using the UniCel DxH 800 analyzer. The SII level was determined by multiplying the platelet count by the neutrophil count/lymphocyte count. SII was designed as the exposure variable in our study.

### 2.4 Covariates

This study included covariates that may impact the relationship between SII and anemia, including gender, age, race/ethnicity (non-Hispanic white, non-Hispanic black, Mexican American, or other races), education level (less than 9 years, 9 to 12 years, more than 12 years), marital status (married or living with a partner, living alone), poverty-to-income ratio (PIR), body mass index (BMI), white blood cell (WBC) counts, smoking status (non-active smoker, active smoker), drinking status (light, moderate, excessive alcohol consumption), menopausal status in female and chronic medical diseases including kidney disease, diabetes, hypertension, stroke, coronary heart disease (CHD), thyroid disease and cancer. The determination of chronic medical diseases was based on the inquiry in the questionnaire of whether the doctor had been informed of the condition in the past.

### 2.5 Statistical analysis

All of the statistical analyses were performed by the Free Statistics software version 1.8 (15) and the statistical software packages R 4.3.2 (http://www.R-project.org, The R Foundation). Mean ± standard deviation, and frequencies (percentages) were used to describe demographic and clinical data. Differences between the general characteristics were tested using the t-test and chi-square test.

Univariate and multivariate logistic regression were used to examine the association between SII and anemia. In multivariate logistic regression, SII was analyzed as a continuous variable or tertile categorical variables, with model 1 adjusting for age, gender, race and model 2 further adjusting for education, marital status, PIR, BMI, smoking status, drinking status and model 3 further adjusting for WBC counts and chronic medical diseases. Odds ratios (ORs) and 95% confidence intervals (CIs) were computed and P-value<0.05 was considered statistically significant. It was noted that SII values were natural log-transformed (ln-SII) when conducting regression analysis because they were right-skewed distributed. In addition, we performed restricted cubic spline (RCS) regression to explore the dose-response association between SII and anemia. We also analyzed the association between anemia severity and SII using multicategory logistic regression.

Subgroup analysis on the associations of SII with anemia was conducted with stratified factors including gender (male and female), age (<60 and ≥60), PIR (low and median or high), BMI (<25 and ≥25), hypertension (no and yes), kidney disease (no and yes), cancer (no and yes).The study also obtained *P* values for interaction in these groups. At the same time, RCS stratified by gender was also conducted. In female, we further did a subgroup analysis based on menopausal status (non-menopausal and menopause) and new age range (<45 and ≥45). Sensitivity analyses were performed by excluding participants with high or low platelet counts (<100 or >450×10^3^ cells/μl). The exclusion criteria for our study refer to two references related to thrombocytopenia (16) and thrombocytosis (17).

## 3 Results

### 3.1 Baseline characteristics of the study population

**Figure 1:**
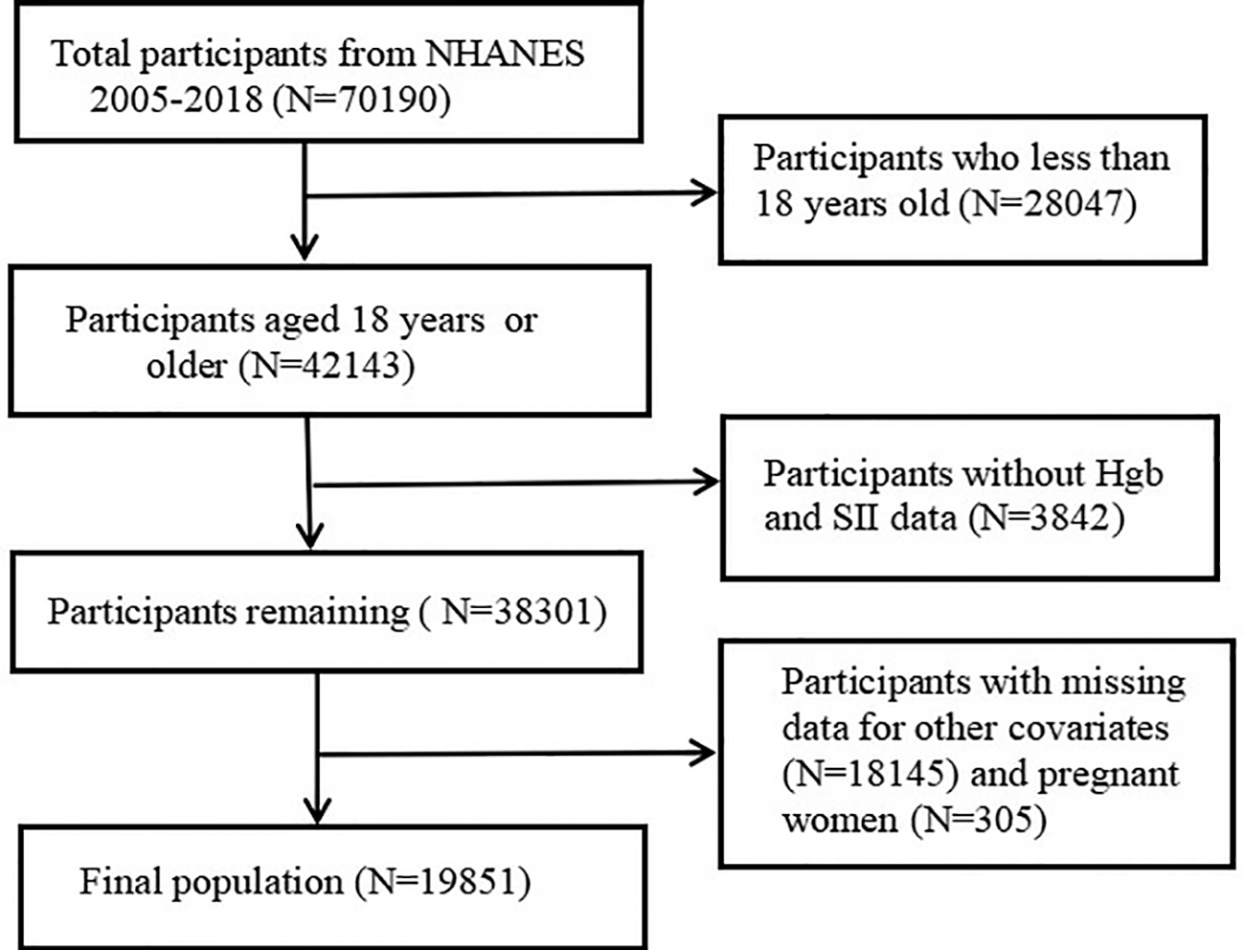
Flowchart of the study population. NHANES, National Health and Nutrition Examination Survey; Hgb, Hemoglobin; SII, systemic immune-inflammation index.

In total, 70,190 participants completed the interview, of whom 28,047 participants were less than 18 years old. Participants without Hgb values and SII data (n=3842) were excluded. We excluded those missing data on other covariates (n = 18,145) and pregnant women (n = 305). Ultimately, this cross-sectional study included 19,851 participants from the NHANES between 2005 and 2018 in the analysis. The detailed inclusion and exclusion process is shown in **Figure 1**.

Of the 19,851 participants included in the study, 10651 (53.7%) were male and 9,200 (46.3%) were female, with an average age of 47.0 ± 17.0. The prevalence of anemia was 7.6% in all participants. Compared with the non-anemia group, those with anemia were more likely to be non-Hispanic black, living alone, lower PIR, higher BMI, non-active smoker, light alcohol consumption, a higher prevalence of chronic medical disease (kidney disease, diabetes, hypertension, stroke, CHD, thyroid disease and cancer) and higher SII (all p<0.001). The clinical and biochemical characteristics of the participants according to anemia are shown in **Table 1**.

**Table 1:** Population characteristics by presence of anemia.

Characteristic	Total	Non-anemia	Anemia	P-Value
NO.	19851	18350	1501	
Age(year), Mean ± SD	47.0± 17.0	46.6 ± 16.8	52.1 ± 18.0	< 0.001
Gender,n(%)				< 0.001
Male	10651 (53.7)	10092 (55)	559 (37.2)	
Female	9200 (46.3)	8258 (45)	942 (62.8)	
Race/ethnicity,n(%)				< 0.001
Non-Hispanic white	9320 (46.9)	8856 (48.3)	464 (30.9)	
Non-Hispanic black	3920 (19.7)	3280 (17.9)	640 (42.6)	
Mexican American	2908 (14.6)	2743 (14.9)	165 (11)	
Others	3703 (18.7)	3471 (18.9)	232 (15.5)	
Education level (year), n (%)				0.128
< 9	1277 (6.4)	1172 (6.4)	105 (7)	
9–12	6873 (34.6)	6325 (34.5)	548 (36.5)	
>12	11701 (58.9)	10853 (59.1)	848 (56.5)	
Marital status, n (%)				< 0.001
Married or living with a partner	12008 (60.5)	11180 (60.9)	828 (55.2)	
Living alone	7843 (39.5)	7170 (39.1)	673 (44.8)	
PIR, Mean ± SD	2.7 ± 1.7	2.8 ± 1.7	2.5 ± 1.6	< 0.001
BMI(kg/m^2^), Mean ± SD	29.0 ± 6.9	29.0 ± 6.8	29.9 ± 8.1	< 0.001
WBC(x10^9^/L), Mean ± SD	7.2 ± 3.7	7.2 ± 2.3	7.2 ± 10.8	0.808
Smoking status, n (%)				< 0.001
Non-active smoker	10090 (50.8)	9239 (50.3)	851 (56.7)	
Active smoker	9761 (49.2)	9111 (49.7)	650 (43.3)	
Drinking status, n (%)				< 0.001
Light alcohol consumption	6983 (35.2)	6300 (34.3)	683 (45.5)	
Moderate alcohol consumption	8360 (42.1)	7744 (42.2)	616 (41)	
Excessive alcohol consumption	4508 (22.7)	4306 (23.5)	202 (13.5)	
Kidney disease, n (%)				< 0.001
No	19409 (97.8)	18007 (98.1)	1402(93.4)	
Yes	442 (2.2)	343 (1.9)	99 (6.6)	
Diabetes, n (%)				< 0.001
No	17924 (90.3)	16710 (91.1)	1214(80.9)	
Yes	1927 (9.7)	1640 (8.9)	287(19.1)	
Hypertension, n (%)				< 0.001
No	13449 (67.7)	12638 (68.9)	811 (54)	
Yes	6402 (32.3)	5712 (31.1)	690 (46)	
CHD, n (%)				< 0.001
No	19183 (96.6)	17784 (96.9)	1399(93.2)	
Yes	668 (3.4)	566 (3.1)	102 (6.8)	
Stroke, n (%)				< 0.001
No	19330 (97.4)	17911 (97.6)	1419(94.5)	
Yes	521 (2.6)	439 (2.4)	82 (5.5)	
Thyroid disease, n (%)				< 0.001
No	18081 (91.1)	16757 (91.3)	1324(88.2)	
Yes	1770 (8.9)	1593 (8.7)	177 (11.8)	
Cancer, n (%)				< 0.001
No	18107 (91.2)	16804 (91.6)	1303(86.8)	
Yes	1744 (8.8)	1546 (8.4)	198 (13.2)	
SII(x10^9^/L), Mean ± SD	531.3 ± 320.6	525.9 ± 312.0	597.7 ± 405.5	< 0.001

PIR, poverty-to-income ratio; BMI, body mass index; CHD, coronary heart disease; SII, systemic immune-inflammation index.

### 3.2 Relationship between SII and anemia

The results of the multifactorial logistic regression analysis showed that SII was positively related to the risk of anemia. This association was significant both in our crude model and partially adjusted model (model 1 and model 2). After adjusting for all covariates in model 3, the positive association between SII and anemia remained stable (OR = 1.51; 95% CI, 1.36–1.68, p<0.001), indicating that each unit increase in ln-SII increased the likelihood of having anemia by 51%. In a sensitivity analysis, a fully adjusted model for the SII tertile indicated a stable positive relationship between SII and anemia. Compared with the lowest SII tertile, participants in the highest SII tertile had a 68% increased risk of developing anemia (OR=1.68; 95% CI, 1.46–1.93, p<0.001). Participants in the middle SII tertile also show a higher risk of anemia compared with the lowest tertile, while this association did not meet the statistical significance. The *p* value for trend of the four models were all <0.001 (**Table 2**).

**Table 2:** Odds ratios (95% confidence intervals) for the association between SII and anemia in different models.

	OR(95% CI), P-Value			
	Crude model	Model 1	Model 2	Model 3
Continuous	1.28 (1.16~1.42), <0.001	1.54 (1.39~1.70), <0.001	1.55 (1.4~1.72), <0.001	1.51 (1.36~1.68), <0.001
Categories				
Tertile 1	Reference	Reference	Reference	Reference
Tertile 2	0.91 (0.79~1.04), 0.153	1.06 (0.92~1.23), 0.384	1.07 (0.93~1.24), 0.330	1.07 (0.92~1.23), 0.378
Tertile 3	1.38 (1.21~1.56), <0.001	1.7 (1.49~1.94), <0.001	1.72 (1.5~1.96), <0.001	1.68 (1.46~1.93), <0.001
P for Trend	<0.001	<0.001	<0.001	<0.001

In sensitivity analysis, SII was converted from a continuous variable to a categorical variable (tertiles).

OR, odds ratio; 95% CI, 95% confidence interval.

Crude model: unadjusted.

Model 1: adjusted for gender, age, race.

Model 2: adjusted for gender, age, race, education, marital status, income, BMI, smoking status, drinking status.

Model 3: adjusted for gender, age, race, education, marital status, income, BMI, smoking status, drinking status, WBC, Chronic medical diseases (include hypertension, coronary heart disease, stroke, diabetes mellitus, kidney disease, thyroid disease and cancer).

**Figure 2:**
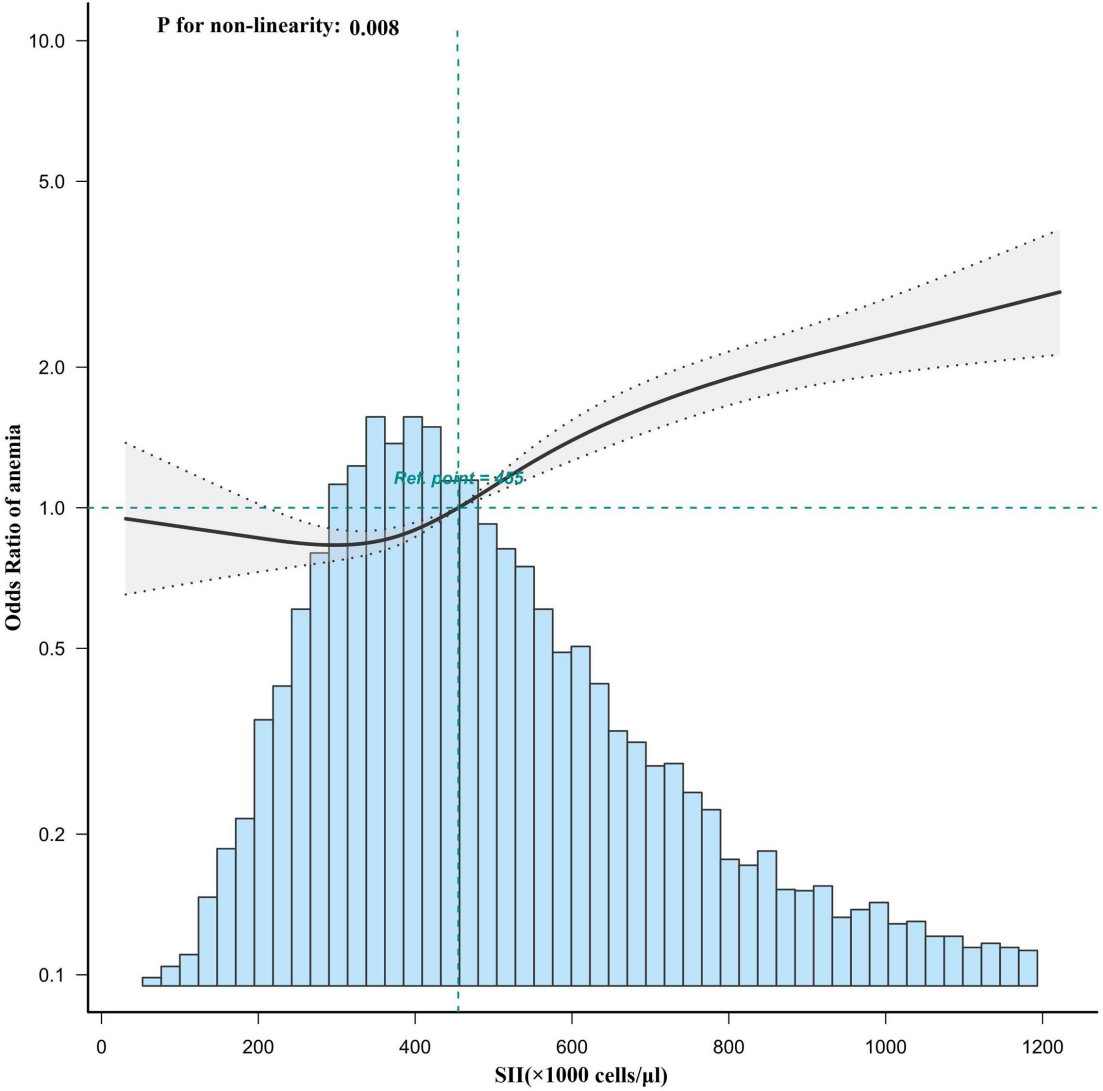
Association between SII and anemia odds ratio. Solid and dashed lines represent the predicted value and 95% confidence intervals. SII, systemic immune-inflammation index.

The restricted cubic spline regression in the fully adjusted analyses revealed that SII was related to risk of anemia in a nonlinear manner (P for nonlinearity = 0.008) (**Figure 2**). Moreover, a threshold effect can be observed, with an inflection point at the SII value of 455 which means a rapidly increasing risk of anemia as the SII value exceeded the cutoff value.

The results of the multicategory logistic regression analysis showed that the more severe the anemia, the stronger the positive correlation between SII and anemia. Compared with no anemia group, the OR values for SII and anemia in mild anemia group were (OR=1.42; 95% CI, 1.24–1.61, p<0.001) and in moderate or severe anemia group were (OR=2.27; 95% CI, 1.88–2.74, p<0.001) after adjusting for all covariates (**Table 3**).

**Table 3:** Association between SII and anemia of varying severity.

	OR(95% CI), P-Value			
	Crude model	Model 1	Model 2	Model 3
NO anemia	Reference	Reference	Reference	Reference
Mild anemia	1.10 (0.98~1.24), 0.089	1.32 (1.18~1.48), <0.001	1.33 (1.19~1.49), <0.001	1.42 (1.24~1.61), <0.001
Moderate or severe anemia	1.95 (1.62~2.35), <0.001	2.35 (1.94~ 2.84), <0.001	2.38 (1.96~ 2.88), <0.001	2.27 (1.88~ 2.74), <0.001

Anemia was divided into no anemia group, mild anemia group, moderate or severe anemia group according to the Hgb values.

OR, odds ratio; 95% CI, 95% confidence interval.

Crude model: unadjusted.

Model 1: adjusted for gender, age, race.

Model 2: adjusted for gender, age, race, education, marital status, income, BMI, smoking status, drinking status.

Model 3: adjusted for gender, age, race, education, marital status, income, BMI, smoking status, drinking status, WBC, Chronic medical diseases (include hypertension, coronary heart disease, stroke, diabetes mellitus, kidney disease, thyroid disease and cancer).

### 3.3 Subgroup analyses and sensitivity analyses

**Figure 3:**
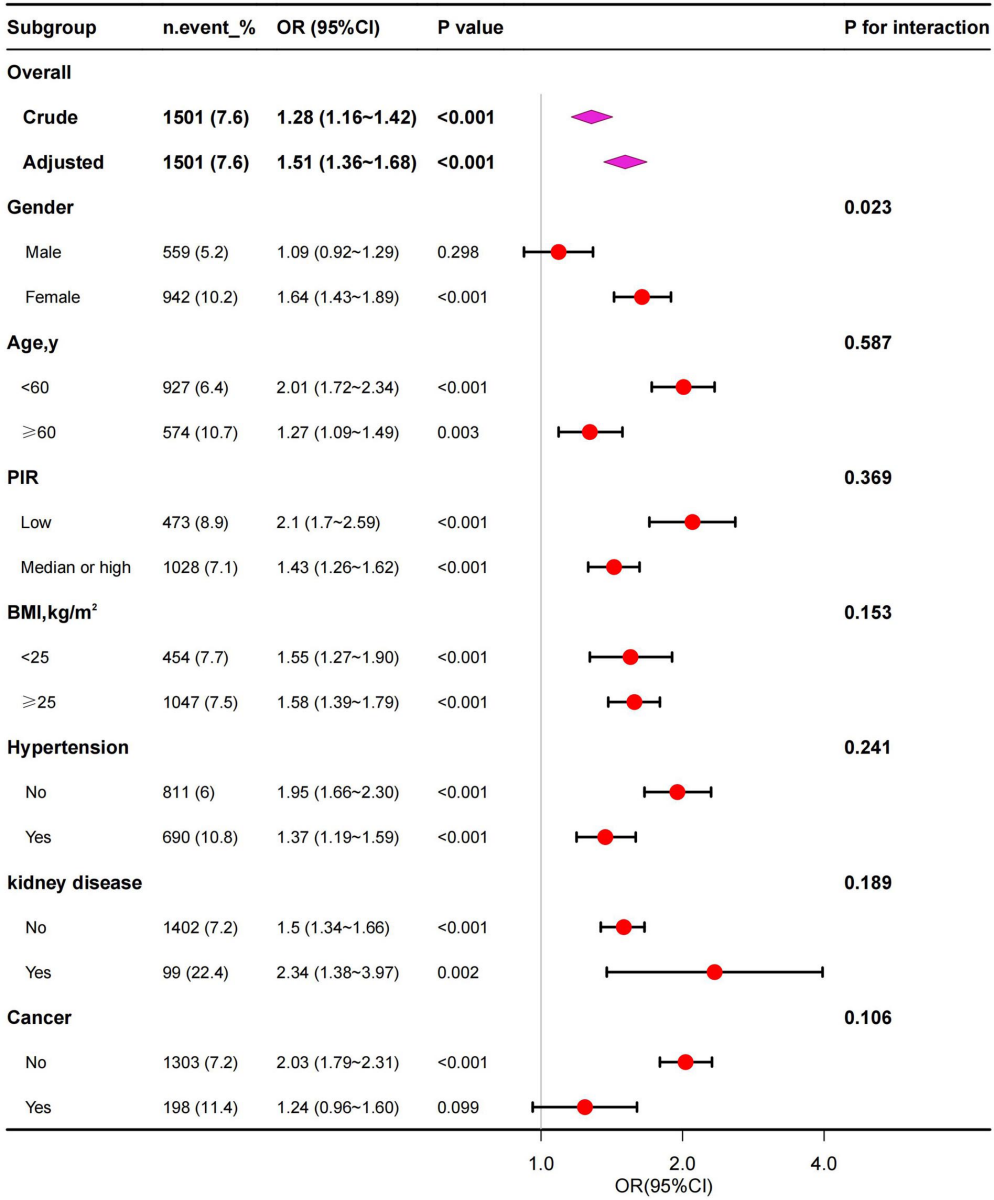
Subgroup analysis for the association between SII and anemia. PIR, poverty-to-income ratio; BMI, body mass index.

Subgroup analyses were performed to assess potential effect modifications on the associations between SII and anemia. A significant association of SII with anemia was observed in each subgroup for the subgroup stratified by age, PIR, BMI, hypertension and kidney disease (all p<0.01). As for the subgroup stratified by gender and cancer, connection with statistical significance was only observed among female and without cancer participants. Although not statistically significant, a positive association between SII and anemia was observed in male and with cancer participants. The interaction test revealed no significant differences among age, PIR, BMI, hypertension, kidney disease and cancer in the relationship between SII and anemia, demonstrating that these factors had no significant influence on this positive relationship (p for interaction >0.05). On the contrary, gender may influence the positive association between SII and anemia (p for interaction <0.05) (**Figure 3**).

Furthermore, we compared the gender differences on the positive correlation between SII and anemia using stratified fitting curves which showed that the positive correlation was stronger in female than in male (**Supplementary Figure S1**). Because the positive correlation was stronger in female, we further analyzed the effect of menopausal status and age range on this relationship. The results showed that the positive association was stronger in the non-menopausal group and in the younger group (age<45) for female (**Table 4**).

**Table 4:** Subgroup analysis of the association between SII and anemia according menopausal status and age range in female. .

Subgroup	n.event_%	OR(95%CI)	P value	P for interaction
Stratified by menopausal status				0.546
Non-menopausal	636(12.9)	2.05 (1.67~2.52)	<0.001	
Menopause	219(6.8)	1.87 (1.39~2.51)	<0.001	
Stratified by age				0.077
<45	519 (11.6)	2.03 (1.62~2.55)	<0.001	
≥45	423 (8.9)	1.75 (1.45~2.12)	<0.001	

Subgroup analysis based on menopausal status (non-menopausal and menopause) and age range (<45 and ≥45).

OR, odds ratio; 95% CI, 95% confidence interval.

To support our conclusions, we conducted sensitivity analyses. After excluding the individuals with abnormal platelet counts, 890 individuals left, and the association between SII and anemia remained stable (**Supplementary Table S1**).

## 4 Discussion

The role of the inflammatory response in the development and progression of anemia is an increasingly studied area in recent years. In this nationally representative cross-sectional study, we observed that participants with higher SII showed an increased likelihood of anemia. Moreover, we found a nonlinear relationship between SII levels and the risk of anemia after adjusting for all covariates. We further found that when SII is higher than 455, the risk of anemia will increase significantly. Additionally, as anemia becomes more severe, the positive correlation between SII and anemia becomes stronger. Subgroup analyses showed that there were stratification effects in female participants. Sensitivity analyses showed that our results remain robust. As far as we know, this is the first study to investigate the relationship between SII and the risk of anemia by using a representative sample of US adults.

SII was first developed in 2014 and was analyzed for its prognostic value in patients with hepatocellular carcinoma (9). SII was calculated by counting three kinds of circulatory cells: neutrophils, lymphocytes, and platelets. As a novel systemic immune inflammation index, there have been no studies on the association between anemia and SII in the previous literature. However, anemia has been linked in many clinical studies with several traditional inflammatory indicators. Current research on clinical inflammatory factors affecting anemia mainly focuses on NLR, immune cells, cytokines and C-reactive protein (CRP) (10). NLR is emerging as an indicator of systemic inflammation. Yazeed et al. demonstrated that in a large number of Saudi subjects, anemia group exhibited a significant increase in NLR compared to the non-anemic group (18). Another study also showed NLR was significantly higher in anemics (19). A multicenter prospective cohort study of 4955 elderly Chinese cancer patients (aged ≥65 years) suggested that a high NLR was an independent risk factor for anemia in older patients with cancer (20). Inflammatory cytokines, such as interleukin-6 (IL-6), interleukin-1 (IL-1), and interferons are signaling molecules that play a key role in the inflammatory response and have been implicated in the development of anemia (21). Macciò et al. reported that IL-6 negatively correlated with Hgb level and was an independent factor for anemia in ovarian cancer (22). CRP is an acute phase protein produced in response to inflammation, and elevated levels of CRP have been associated with anemia and can serve as a marker for inflammation related anemia (23, 24). These anemia-associated inflammatory factors are produced by activated immune cells or other cells stimulated by inflammation and serve as important mediators of the immune and inflammatory processes. Meanwhile, cytokines in the inflammatory response activate immune cells such as lymphocytes, granulocytes and macrophages to participate in the immune response (25). Platelets, while primarily known for their role in blood clotting, also have immune-modulatory functions and can release inflammatory mediators in response to various stimuli (26).

Most study findings about the relationship between above inflammatory parameters and anemia are consistent with our findings. Compared to traditional inflammatory factors, the SII selected in this study included more clinical information than one or two kinds of peripheral blood markers could truly reflect the burden of inflammation. Several studies showed that SII has been confirmed the better prognostic value in many diseases compared with other inflammatory factors (7–9, 27). Ling et al. found that SII had better predictive performance compared with other inflammatory factors in the early prediction of acute kidney injury in severe acute pancreatitis patients (8). Another study reported that SII was more accurate and effective in predicting the outcomes of patients with cervical cancer compared with NLR, platelet/lymphocyte ratio (PLR), and monocyte/lymphocyte ratio (MLR) (7). Similar results were confirmed in some other studies such as esophageal squamous cell carcinoma (27), hepatocellular carcinoma (9). Additionally, SII had been shown to be associated with a wide range of diseases and medical conditions including kidney stones (28), diabetic depression (29), coronary heart disease (30), heart failure (31), asthma (32), rheumatoid arthritis (33), hepatic steatosis (34). For example, Xingpeng et al. showed SII was positively associated with a high risk of kidney stones in US adults aged less than 50 (28). Therefore, as a reliable and minimally invasive biomarker, SII has promising prospects for clinical application.

The exact mechanisms underlying the positive association between inflammation and anemia still remain unclear (35). Inflammation can disrupt the normal processes of red blood cell production, iron metabolism, and erythropoiesis, leading to anemia (11). Inflammation, particularly chronic inflammation, can lead to the release of pro-inflammatory cytokines such IL-6 and tumor necrosis factor alpha (TNF-a). These cytokines can directly inhibit the production and maturation of red blood cells in the bone marrow, disrupting erythropoiesis and leading to reduced red blood cell counts (13). Furthermore, inflammation can alter iron metabolism by promoting the sequestration of iron within cells and reducing its availability for erythropoiesis. This can lead to functional iron deficiency, where iron levels appear normal in the body, but are not effectively utilized for red blood cell production (36). Additionally, inflammatory processes can lead to the dysregulation of erythropoietin, a hormone critical for the production of red blood cells. This can further impair the body’s ability to respond to anemia by producing adequate numbers of red blood cells (37).

In this study, stratified analysis showed that the positive association between SII and anemia was statistically significant only for females and not for males. The observed gender differences could potentially be attributed to the differential immune responses mounted by inflammatory cells in males and females (38). It is well-documented that females generally exhibit stronger and faster innate and adaptive immune responses compared to males. Sex hormones, particularly estrogen, contribute to the development and activity of the immune system. Both innate and adaptive immune systems have receptors for sex hormones and respond to hormonal cues accounting for differences in gender-related immune responses (39). Additionally, females of reproductive age are more susceptible to anemia due to factors such as blood loss during menstruation, childbirth, and hemodilution during pregnancy (40, 41). Compared to postmenopause, females in non-menopausal had lower iron stores (42). Inflammation is associated with anemia by affecting iron metabolism, as described in the above mechanism description. Our study also found a higher likelihood of anemia and a stronger positive association between SII and anemia in non-menopausal and younger female. These gender-specific factors may contribute to the observed disparity in the relationship between SII and anemia.

This research explored the association between SII and anemia and provided several advantages. Firstly, we used a nationally representative and large enough sample of adults in the US. Secondly, we adjusted for known and potential variables, such as WBC counts and chronic medical diseases to produce more reliable results. Additionally, we used restrictive cubic spline and smooth curve fitting to explore their non-linear relationship. Finally, to confirm the robustness of our results, we conducted sensitivity analyses on platelet which was a parameter of SII and had a definite effect on anemia (43).

However, this research also has limitations that should be considered. First, due to the cross-sectional study design, we could not obtain causal inferences and prospective studies are required to elucidate the causality. Second, Hgb values and the cell counts were obtained from one blood test only. Serial testing may be more reliable. Third, although some potential covariates were adjusted, the influence of other possible confounding factors could not be completely excluded.

## 5 Conclusion

This cross-sectional study demonstrated that SII was positively associated with the risk of anemia especially among female participants. Further large-scale prospective studies are still needed to analyze the role of SII in anemia.

## Data availability statement

The original contributions presented in the study are included in the article/**Supplementary Materials**, further inquiries can be directed to the corresponding author/s.

## Ethics statement

The studies involving humans were approved by National Center for Health Statistics (NCHS) Ethics Review Committee. The studies were conducted in accordance with the local legislation and institutional requirements. The participants provided their written informed consent to participate in this study.

## Author contributions

SC: Data curation, Writing – original draft. JX: Writing – review & editing, Data curation. WC: Data curation, Writing – review & editing. XL: Writing – review & editing. CL: Writing – review & editing. YD: Writing – original draft. YZ: Writing – original draft. GS: Writing – original draft. QS: Writing – review & editing. HW: Writing – review & editing. ZX: Project administration, Writing – review & editing.
