# Supplementary material for: Association of the systemic immune-inflammation index with anemia: a population-based study
Source: Front Immunol. 2024 May 10;15:1391573. doi: 10.3389/fimmu.2024.1391573 (PMC11116595; doi:10.3389/fimmu.2024.1391573)
Supplement: Supplementary file 1 [file DataSheet_1.pdf]

Supplementary Material

Figure S1. The relationship between SII and anemia stratified by gender.

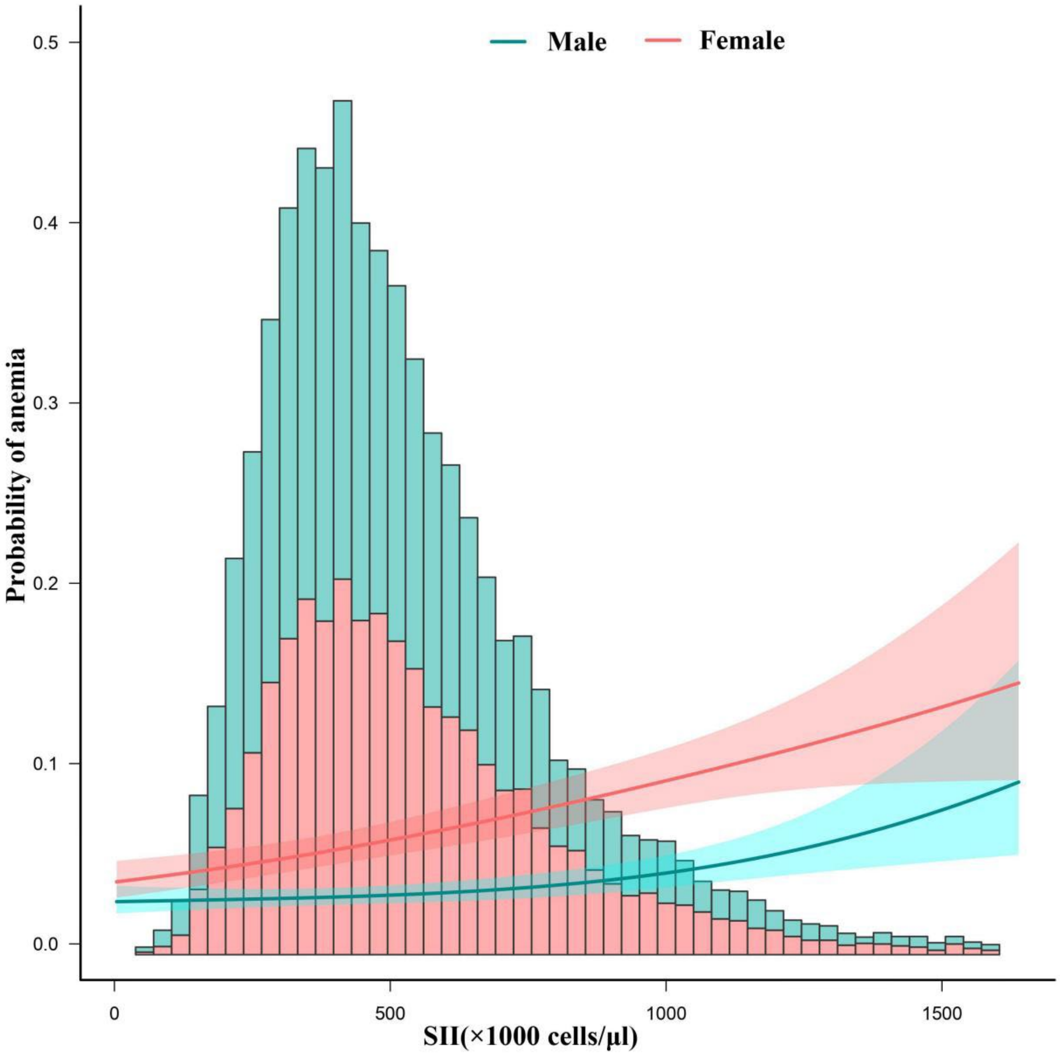

SII, systemic immune-inflammation index.

Table S1. Sensitivity analyses on platelet.

	OR(95% CI), P-Value			
	Crude model	Model 1	Model 2	Model 3
Continuous	1.35 (1.21~1.5), <0.001	1.65 (1.48~1.84), <0.001	1.67 (1.49~1.86), <0.001	1.72 (1.52~1.96), <0.001
Categories				
Tertile 1	Reference	Reference	Reference	Reference
Tertile 2	0.96 (0.83~1.11), 0.598	1.14 (0.98~1.32), 0.093	1.14 (0.98~1.33), 0.079	1.15 (0.98~1.33), 0.078
Tertile 3	1.38 (1.21~1.58), <0.001	1.72 (1.5~1.98), <0.001	1.74 (1.51~2.00), <0.001	1.74 (1.49~2.04), <0.001
P for Trend	<0.001	<0.001	<0.001	<0.001

SII was converted from a continuous variable to a categorical variable (tertiles).
OR, odds ratio; 95% CI, 95% confidence interval.
Crude model: unadjusted
Model 1: adjusted for gender, age, race.
Model 2: adjusted for gender, age, race, education, marital status, income, BMI, smoking status, drinking status.
Model 3: adjusted for gender, age, race, education, marital status, income, BMI, smoking status, drinking status, WBC, Chronic medical diseases (include hypertension, coronary heart disease, stroke, diabetes mellitus, chronic kidney disease, thyroid disease and cancer).
